# MiR-302b as a Combinatorial Therapeutic Approach to Improve Cisplatin Chemotherapy Efficacy in Human Triple-Negative Breast Cancer

**DOI:** 10.3390/cancers12082261

**Published:** 2020-08-12

**Authors:** Alessandra Cataldo, Sandra Romero-Cordoba, Ilaria Plantamura, Giulia Cosentino, Alfredo Hidalgo-Miranda, Elda Tagliabue, Marilena V. Iorio

**Affiliations:** 1 Molecular Targeting Unit, Fondazione IRCCS Istituto Nazionale dei Tumori of Milan, 20133 Milan, Italy; alessandra.cataldo@istitutotumori.mi.it (A.C.); sromero_cordoba@hotmail.com (S.R.-C.); ilaria.plantamura@istitutotumori.mi.it (I.P.); giulia.cosentino@istitutotumori.mi.it (G.C.); elda.tagliabue@istitutotumori.mi.it (E.T.); 2 Biochemistry Department, Instituto Nacional de Ciencias Médicas y Nutrición Salvador Zubirán, Mexico City 14080, Mexico; 3 Cancer Genomics Laboratory, National Institute of Genomic Medicine, Mexico City 14610, Mexico; ahidalgo@inmegen.gob.mx

**Keywords:** microRNAs, triple-negative breast cancer, cisplatin, drug response

## Abstract

*Introduction*: Chemotherapy is still the standard of care for triple-negative breast cancers (TNBCs). Here, we investigated miR-302b as a therapeutic tool to enhance cisplatin sensitivity in vivo and unraveled the molecular mechanism. *Materials and Methods*: TNBC-xenografted mice were treated with miR-302b or control, alone or with cisplatin. Genome-wide transcriptome analysis and independent-validation of Integrin Subunit Alpha 6 (ITGA6) expression was assessed on mice tumor samples. Silencing of ITGA6 was performed to evaluate cisplatin response in vitro. Further, potential transcription factors of ITGA6 (E2F transcription facor 1 (E2F1), E2F transcription factor 2 (E2F2), and Yin Yang 1 (YY1)) were explored to define the miRNA molecular mechanism. The miR-302b expression was also assessed in TNBC patients treated with chemotherapy. *Results*: The miR–302b-cisplatin combination significantly impaired tumor growth versus the control through indirect ITGA6 downregulation. Indeed, ITGA6 was downmodulated in mice treated with miR-302b–cisplatin, and ITGA6 silencing increased drug sensitivity in TNBC cells. In silico analyses and preclinical assays pointed out the regulatory role of the E2F family and YY1 on ITGA6 expression under miR-302b–cisplatin treatment. Finally, miR-302b enrichment correlated with better overall survival in 118 TNBC patients. *Conclusion*: MiR-302b can be exploited as a new therapeutic tool to improve the response to chemotherapy, modulating the E2F family, YY1, and ITGA6 expression. Moreover, miR-302b could be defined as a new prognostic factor in TNBC patients.

## 1. Introduction

MicroRNAs (miRNAs) are a class of small non-coding regulatory RNAs (19–22 nt) that play key roles in different biological processes including cancer [1]. MicroRNAs bind to their mRNA targets and inhibit translation or mediate mRNA degradation. Each miRNA can target a large number of genes [2]. Recently, it has been demonstrated that miRNAs can serve as cancer biomarkers [1] and they are also being proposed to develop therapeutic interventions [3]. MicroRNAs are also able to regulate mechanisms of resistance to chemotherapy such as DNA repair, cell cycle regulation, and escape from apoptotic pathways [4]. Intrinsic or acquired chemotherapy resistance is a relevant feature in the management of neoplastic diseases [5] and is a critical determinant of long-term prognosis in breast cancer [6]. Of particular interest, triple-negative breast cancer (TNBC) accounts for 15% to 20% of all breast cancer cases and is characterized by poor clinical outcome. Targeted therapies are not currently available in first-line regimens for this tumor subtype [7], and most patients still mainly depend on conventional chemotherapy such as taxanes and anthracyclines [8]. In the last years, there has been a renewed interest in cisplatin-based treatment, particularly for BReast CAncer gene (BRCA)-deficient TNBCs, that show a greater susceptibility to DNA-damaging chemotherapy agents, which can be used alone or in combination [9]. Usually, TNBC patients show a good initial response to cisplatin-based chemotherapy but often chemoresistance occurs [10], reducing the clinical efficacy of the drug [11]. However, the molecular mechanisms involved in chemotherapy resistance of TNBC have not been completely elucidated. Recently, we showed that miR-302b overexpression enhances sensitivity to cisplatin in breast cancer cell lines, reducing cell viability and proliferation in response to the treatment [12]. MicroR-302b belongs to the miR-302 family, which includes miR-302a, -302a*, -302b, -302b* -302c, -302c*, -302d, -367, and -367* members [13], and is involved in the self-renewal and proliferation of embryonic stem cells [14]. Many studies have demonstrated that miR-302b also has an oncosuppressive role in different types of cancer [15,16,17,18,19]. We have reported that miR-302b directly targets E2F transcription factor 1 (E2F1), a master regulator of the G1/S transition of cell cycle, and indirectly ATM, the main sensor of DNA damage [12]. Thus, miR-302b expression affects cell cycle progression and DNA damage response, enhancing apoptosis activation upon cisplatin treatment [12]. Other groups demonstrated that miR-302 family sensitizes breast cancer cells to radiotherapy and chemotherapy [20,21,22]. In particular Liang et al. [20] demonstrated that lower expression of miR-302a promotes resistance to radiotherapy, and that re-introduction of miR-302a enhances the sensitivity of breast cancer cells to radiotherapy. Moreover, the miR-302 family targets breast cancer resistance protein (BCRP) thus increasing chemosensitivity [21], mainly to doxorubicin compound [22]. Very recently, it was confirmed that miR-302b has a tumor suppressor function in breast cancer, and its reintroduction reduces tumor progression by targeting Runt-related transcription factor 2 (RUNX2). Moreover, the downregulation of miR-302b associates with poor survival in breast cancer patients before chemotherapy treatment [23]. Since we previously demonstrated that miR-302b plays a relevant role in the response of breast cancer cell lines to cisplatin treatment in vitro [12], in this study, we investigated whether miR-302b miRNA-based therapy can be used as a novel tool to enhance the response to cisplatin chemotherapy in a TNBC mouse model. Through in vivo and in vitro preclinical models and genome-wide mRNA profiling, we investigated the mechanism behind miR-302b activity in combination with cisplatin and identified a biological axis involving Integrin Subunit Alpha 6 (ITGA6), Yin Yang 1 (YY1), and the E2F transcription factor family. Finally, we also investigated the potential role of miR-302b as a prognostic biomarker in our TNBC cohort (Ital–Mex). Our results represent the proof of concept for the future potential use of miR-302b in a miRNA-based therapy for TNBC patients treated with cisplatin and as a prognostic factor in these patients.

## 2. Results

### 2.1. MiR-302b Enhances Sensitivity to Cisplatin In Vivo

**Figure 1:**
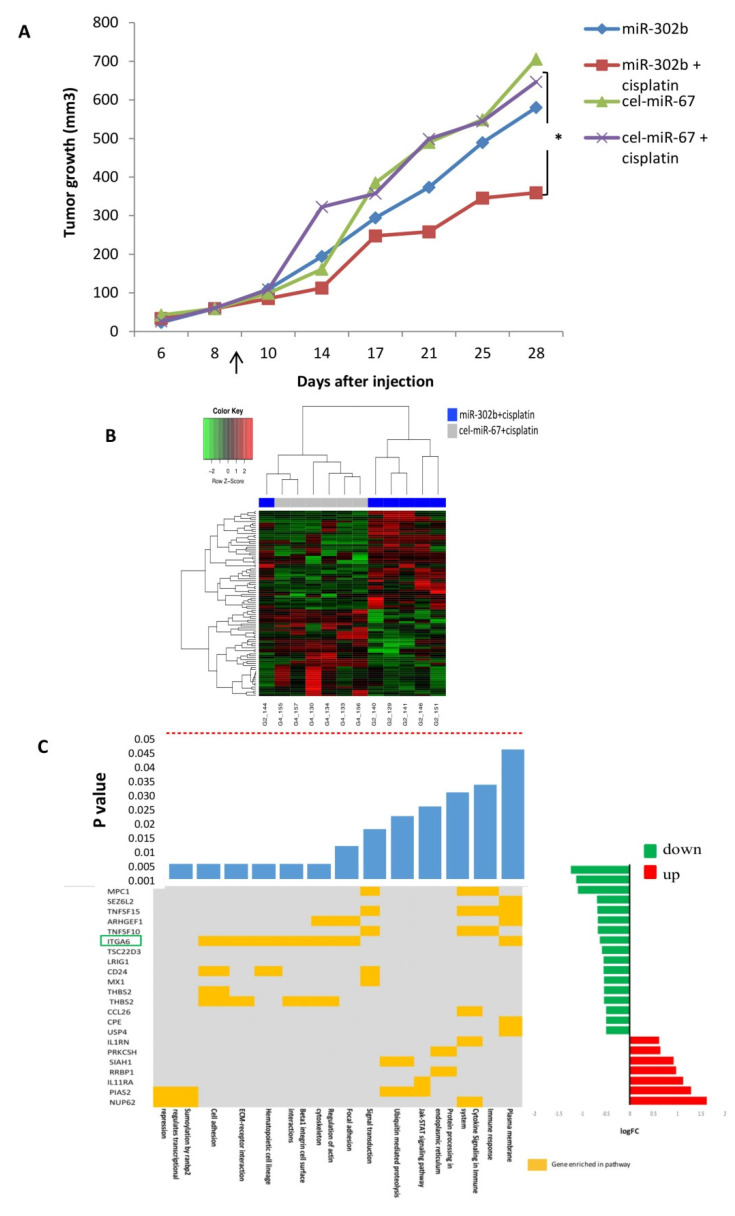
Effects of miR-302b and cisplatin treatment in an orthotopic murine triple-negative breast cancer (TNBC) model and transcriptional landscape established by miR-302b and cisplatin treatment in an in vivo assay. (**A**) Tumor volumes in Severe Combined Immunodeficient (SCID) mice after the peritumoral injection of miR-302b or miR-cel-67 control plus cisplatin or saline treatment evaluated across different time points. Data presented are mean values (*n* = 7; * *p* ≤ 0.05 Student’s *t*-test). Arrow indicates start of treatment. (**B**) Heatmap showing the differentially expressed genes (log fold change > 0.5 and *p* ≤ 0.05) in mice tumors treated with miR-302b mimic plus cisplatin versus cel-miR-67 plus cisplatin. Red or green colors represent up- or down-modulation, respectively; normalized gene expression was adjusted to y–z score. (**C**) Common plot showing gene enrichment analysis based on differently expressed genes. The top bar plot shows the significance of each enriched pathway. Middle panel shows the belonging of a particular gene to any of the enriched pathway (yellow mark). The right bar plot shows the log fold change values of each gene computed by the comparison of mice treated with miR-302b+cisplatin versus mice treated with cel-miR-67 negative control+cisplatin. Red or green colors represent up or down-modulation, respectively. Highlighted gene in green represent the selected candidate for further analysis.

To explore the plausibility of miR-302b miRNA-based therapy in combination with cisplatin to treat TNBC, MDA-MB-231 TNBC cells were orthotopically injected into the mammary fat pad of female Severe Combined Immunodeficient (SCID) mice. Mice were treated with lipid nanoparticles containing miR-302b or cel-miR-67 control, alone or in combination with cisplatin or physiological saline solution. As shown in Figure 1A, tumor growth was significantly reduced in mice receiving the combinatorial treatment with miR-302b and cisplatin, in comparison with mice treated with cel-miR-67 control and cisplatin (*p* = 0.03). Instead, miR-302b treatment alone had no significant effect on tumor growth. To determine whether miR-302b had efficiently been delivered to the tumors, miR-302b expression levels were evaluated. MicroRNA overexpression was confirmed in all miR-302b-treated tissues (Figure S1). These results confirm the potential activity of miR-302b therapy to enhance cisplatin in vivo effect in a TNBC model.

### 2.2. MiR-302b Enhances Cisplatin Response through the Down-Modulation of ITGA6 in Tumor Cells

To analyze the transcriptional landscape established by miR-302b in vivo delivered in combination with cisplatin treatment, a gene-expression profiling of the xenograft tumors from four different experimental groups (i.e., miR-302b, miR-302b+cisplatin, cel-miR-67 control, and cel-miR-67 control+cisplatin) was performed in an Illumina platform. Hierarchical clustering analysis of differentially expressed genes discriminates each experimental condition. When comparing mRNA gene profiling of mice treated with miR-302b and cisplatin versus mice treated with control and cisplatin, 46 genes were down-modulated and 49 upregulated (fold change > 1 and *p* ≤ 0.05) (Figure 1B, Table S1).

**Figure 2:**
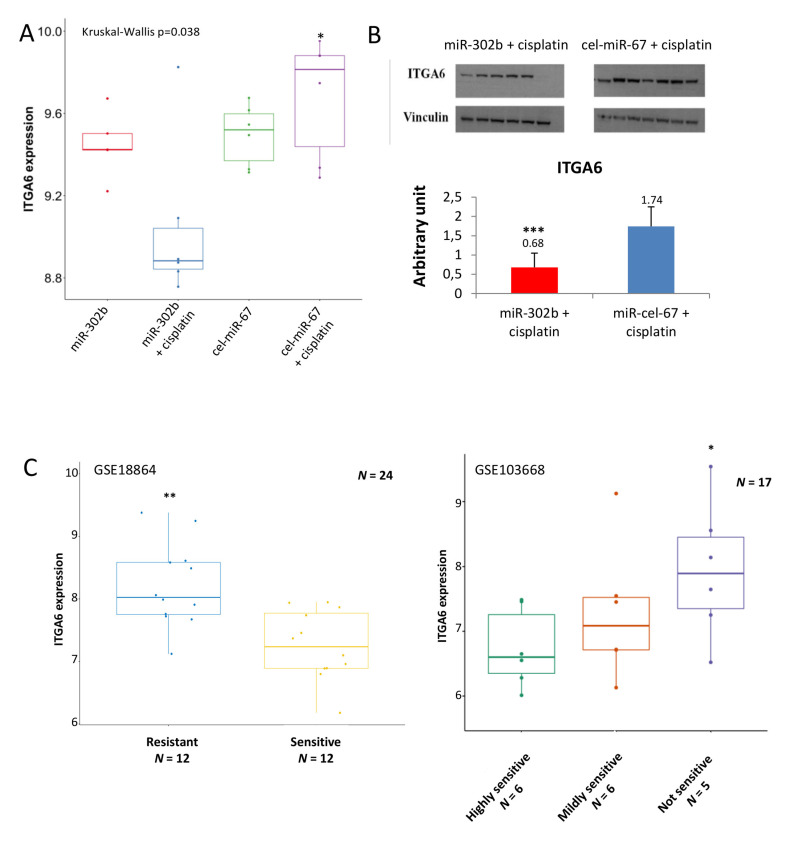
Integrin Subunit Alpha 6 (ITGA6) expression in tumor from mice treated with miR-302b mimic and cisplatin and in public data sets. (**A**) ITGA6 mRNA expression extrapolated from gene expression analysis in each biological group of the in vivo assay (miR-302b mimic; miR-302b mimic+cisplatin; cel-miR-67 negative control; cel-miR-67 negative control+cisplatin) (* *p* ≤ 0.05 miR-302b+cisplatin versus cel-miR-67 negative control+cisplatin). (**B**) Western blot analysis of ITGA6 protein expression in mice tumors treated with miR-302b mimic plus cisplatin and cel-miR-67 plus cisplatin. Upper figure shows specific ITGA6 bands and vinculin bands as housekeeping; lower histogram represents ITGA6 densitometric quantification (*** *p* ≤ 0.001). (**C**) Right panel shows ITGA6 expression in 24 TNBC patients treated in neoadjuvant with cisplatin (GSE18864). Left panel shows ITGA6 expression in additional 17 TNBC patients treated in neoadjuvant with cisplatin (GSE103668). (* *p* ≤ 0.05; ** *p* ≤ 0.001).

To further examine the molecular phenotype of tumors treated in combination with miR-302b and cisplatin, an enrichment pathway analysis was performed with DAVID and Innate DB tools, showing the down-representation of several pathways related to cell adhesion and immune signaling (Figure 1C). These results pointed to ITGA6 as a relevant regulatory target of miR-302b and cisplatin activity. Indeed, ITGA6 mRNA expression was significantly downregulated in tumors treated with miR-302b plus cisplatin compared with other groups (*p* = 0.036) (Figure 2A). We then evaluated ITGA6 protein expression by Western blot analysis confirming its down-modulation in mice treated with miR-302b and cisplatin compared with mice treated with control plus cisplatin (*p* = 0.001) (Figure 2B), although no differential changes were observed when mice were treated with miR-302b alone versus cel-miR-67 alone (Figure S2). Since it is known that high levels of ITGA6 confer resistance to chemotherapy to TNBC cells [24], we performed an in silico analysis in two public data sets (GSE18864 and GSE103668) showing that patients affected with TNBC and non-responsive to neoadjuvant cisplatin treatment have higher ITGA6 expression compared with responders (*p* = 0.001 and *p* = 0.038, respectively) (Figure 2C).

**Figure 3:**
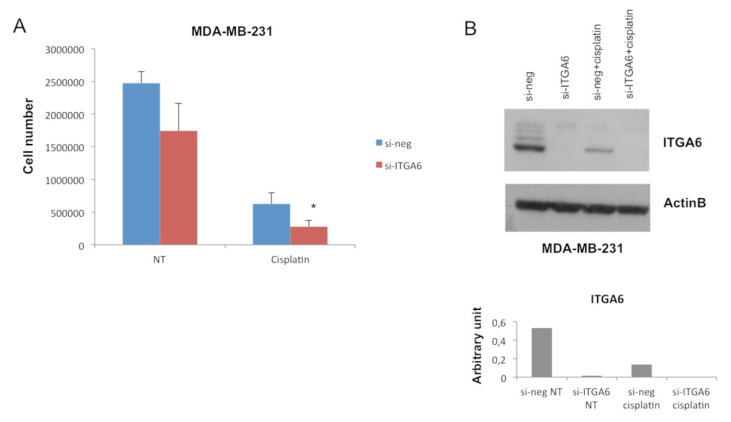
Combinatorial effect of small interfering-ITGA6 (si-ITGA6) and cisplatin in an in vitro TNBC model. (**A**) MDA-MB-231 cell count following si-ITGA transfection and cisplatin treatment compared with their negative controls. (**B**) Western blot analysis of ITGA6 protein expression in MDA-MB-231 cells transfected with si-ITGA6 and treated with cisplatin compared with their negative controls. Upper figure shows specific ITGA6 bands and β-actin bands as housekeeping; lower figure shows histograms representing ITGA6 densitometric quantification. Data are representative of three independent experiments performed at least in triplicate (* *p* ≤ 0.05, Student’s *t*-test).

To demonstrate that ITGA6 down-modulation is involved in the miR-302b-mediated improvement of cisplatin response in TNBC cells, MDA-MB-231 TNBC cells were transiently silenced for ITGA6 using a small-interfering RNA (si-ITGA6) or negative control (si-neg), then treated or not with cisplatin (100 µM), as a phenocopy experiment. Results reveal that ITGA6 silencing significantly enhances sensitivity to cisplatin (*p* = 0.034) (Figure 3A), mimicking the previously observed effect of miR-302b. We confirmed ITGA6 silencing upon si-ITGA6 transfection and a significant ITGA6 down-modulation following cisplatin treatment by western blot analysis (Figure 3B). The same experiment was performed in BT549 TNBC cells, obtaining similar results (*p* = 0.035) (Figure S3). Taken together, these data confirm that miR-302b promotes sensitivity to cisplatin by modulating ITGA6 expression.

### 2.3. Combinatorial Treatment of miR-302b and Cisplatin Downregulates E2Fs Family and YY1, Which in Turn Downregulates ITGA6 Expression

Since ITGA6 is not predicted as a putative target of miR-302b and since miRNA intervention by itself is not sufficient to significantly downregulate ITGA6 (Figure 2A), instead requiring concomitant treatment with cisplatin, we focused our attention on possible cooperative regulatory networks of ITGA6, such as transcription factors (TFs), that might be altered by miR-302b and cisplatin intervention.

**Figure 4:**
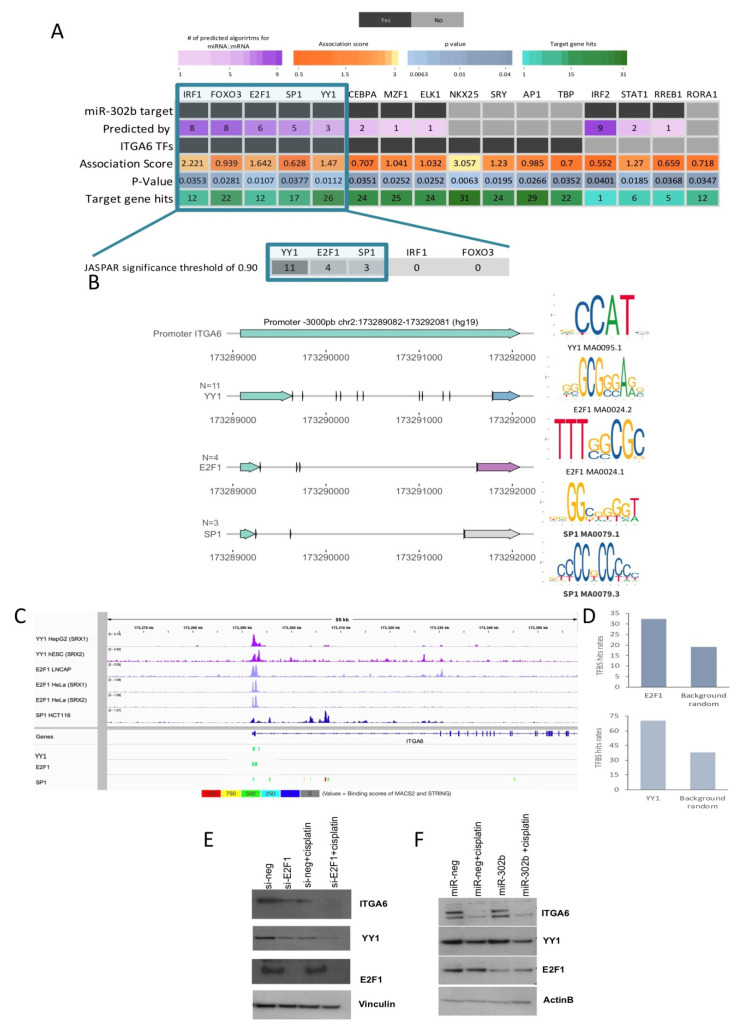
Summary of ITGA6 putative transcription factors (TFs) and their role behind the established mechanism by the combinatorial effect of miR-302b and cisplatin. (**A**) Overview of the study strategy to define possible TFs regulating ITGA6 expression. Through weight TFs matrices enrichment methods of down-modulated genes in the in vivo assay (miR-302+cisplatin versus cel-miR-67 negative control+cisplatin) possible TFs were identified. Only those TFs predicted as miR-302b targets and presenting motif binding sites to the promoter sequence of ITGA6 were considered (highlighted in blue square). Bottom zoom panel shows the top TF candidates and the number of robust bindings sites to ITGA6 promoter computed by JASPAR (significance threshold > 0.9). The square highlights the selected TFs. (**B**) Schematic representation of the robust binding sites of YY1, E2F1, and SP1 on the ITGA6 promoter. Right panel shows the TFs motif from the JASPAR database used in the analysis. (**C**) Representative images of Chip-seq peaks of the selected TFs obtained by ChIP-Atlas and visualized in Integrative Genomics Viewer (IGV). (**D**) Frequency of E2F1 and YY1 TFBS hits among downregulated genes compared to hits in a set of background random genes. ITGA6, YY1, and E2F1 expressions following si-E2F1 knockdown (**E**) and miR-302b transfection (**F**) in MDA-MB-231 cells treated or not with cisplatin. Vinculin and β-actin were used as housekeeping controls. Data are representative of three independent experiments performed at least in triplicate.

We analyzed the downregulated genes resulting from our in vivo analysis between mice treated with concomitant miR-302b and cisplatin versus mice treated with cel-miR-67 and cisplatin to further select the most likely altered TFs that potentially induce ITGA6 and that may significantly contribute to the establishment of the observed expression landscape upon treatment. Thus, the over-represented transcription factor binding-sites on the differentially expressed genes were computed with the oPPOSUM [25] and PASTAA [26,27] algorithms (Figure 4A, Table S2). Only those hits found with both methods were considered for further analysis (Figure S4). We then ranked the resulting common TFs according to their predicted interaction with miR-302b (Figure 4A, Table S2). Only the TFs predicted as miR-302b targets by at least three dedicated algorithms and inferred as feasible ITGA6 TFs by deduced binding motifs were selected (Figure 4A, Table S2). The promoter sequence of ITGA6 (−3000 bp from the transcription start site TSS) was explored with DNA matrices from the JASPAR database [28,29] (Figure 4A,B), using a significance threshold of 0.90 to test the relative enrichment scores of the TFs to ITGA6 target promoter sequence. Yin Yang 1 and E2F1 presented the highest number of robust binding sites on ITGA6 promoter (Figure 4B, Table S3). Using stringent criteria and restricting our analyses to high-confidence scores, E2F1 and YY1 were detected as strong candidates as ITGA6 TFs, supported by public ChIP-seq peak data in different human tissues available on ChIP-Atlas [30] (Figure 4C). Further, the observed binding hits rates (significance threshold matrix of 0.80) were significantly higher than expected by chance based on a random list of genes outside the ITGA6/YY1/E2F1 sub-network (*n* = 21,151) (Figure 4D), suggesting that E2F1 and YY1 contribute to the regulatory transcriptional network established by the combined miR-302b and cisplatin treatment. Thus, mining analysis based on predicted binding profiles and public ChIP-seq data suggest that E2F1 and YY1, known and predicted targets of miR-302b, respectively, could serve as TFs with specific motif sites on the ITGA6 promoter.

**Figure 5:**
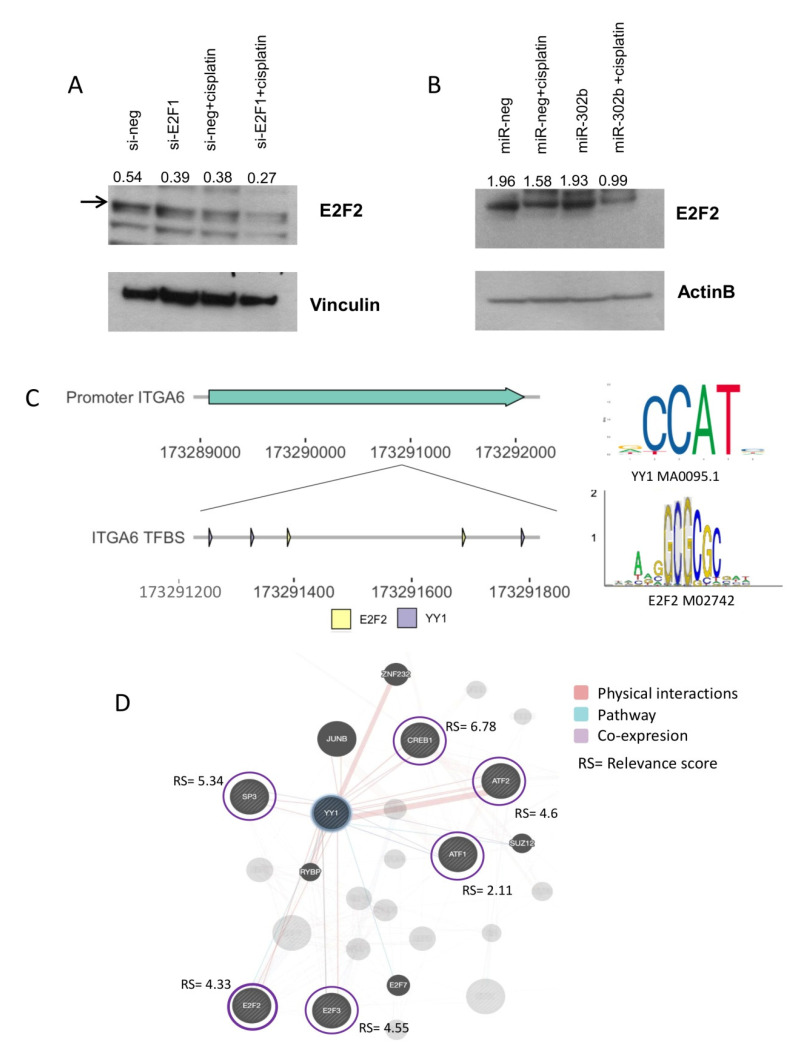
E2F2 protein expression under miR-302b plus cisplatin treatment in an in vitro model and in silico E2F2 correlation with the YY1/ITGA6 biological axis. E2F2 expression after (**A**) si-E2F1 knockdown and (**B**) miR-302b transfection in MDA-MB-231 cells treated or not with cisplatin, respectively. Vinculin and β-actin were used as housekeeping controls. Data are representative of three independent experiments performed at least in triplicate. (**C**) Schematic representation of robust neighbor binding sites of YY1 and E2F2 on the ITGA6 promoter. Right panel shows TFs motif from the JASPAR database used in the analysis. (**D**) Correlation network of YY1 and human TFs predicted to physical interact with YY1.

Further, siRNA-mediated E2F1(si-E2F1) knockdown in MDA-MB-231 TNBC cells, treated or not with cisplatin (100 µM), shows that ITGA6 protein expression was downregulated upon E2F1 silencing or cisplatin administration and, more significantly, following the combined treatment (Figure 4E). Interestingly, YY1, which is a predicted TF of ITGA6, was also downmodulated by E2F1 silencing in a similar way to ITGA6 expression patterns in the different experimental conditions (Figure 4E). The E2F1 silencing was confirmed by Western blot (Figure 4E). These results suggest that ITGA6 and YY1 are directly modulated by E2F1, a well-known target of miR-302b. Paradoxically, when miR-302b was exogenously expressed in MDA-MB-231 TNBC cells, treated or not with cisplatin (100 µM), YY1 and ITGA6 down-modulation was observed only in presence of miR-302b and concomitant cisplatin treatment but not with miR-302b alone, despite what we previously reported with the siRNA-mediated E2F1 knockdown (Figure 4F). Considering the discrepancy between si-E2F1 and miR-302b effects, we hypothesized that another cooperative mechanism among the TF axis and miR-302b occurred. In particular, we hypothesized that other E2F family members, not targeted by miR-302b, could be involved in this mechanism, resulting co-regulated with E2F1 following siRNA-mediated knockdown. Indeed, we analyzed the effect of E2F1 silencing on E2F transcription factor 2 (E2F2) expression, which also resulted downregulated upon E2F1 knockdown; likewise, the combined treatment with cisplatin negatively modulates it (Figure 5A). On the contrary, exogenous expression of miR-302b alone did not lead to E2F2 down-modulation, which was achieved only by combinatorial treatment of miR-302b and cisplatin (Figure 5B). All protein quantifications are reported in the Figure S5.

**Figure 6:**
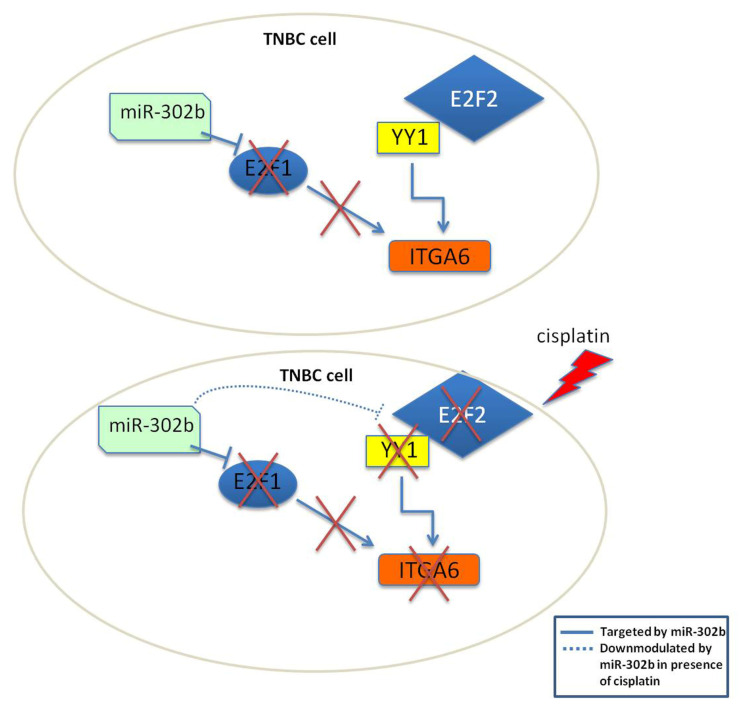
Schematic representation of proposed mechanism of action of miR-302b and cisplatin in TNBC cells. Upper scheme represents the biological activity of miR-302b in monotherapy. Exogenous expression of miR-302b does not affect ITGA6, since YY1 can interact with E2F2 to maintain ITGA6 transcription. Lower scheme represents the effect of combined intervention of miR-302b and cisplatin, that results in YY1 and E2F2 down-modulation impeding their activity over ITGA6 and leading to TNBC cell death. This proposed mechanism acts together with the well-known E2F1 down-modulation, another predicted ITGA6 TF, by miR-302b.

E2F transcription factor 2 is not predicted as a miR-302b target, but there is an evidence that shows E2F2 interaction with YY1 [31]. This was further supported by the fact that E2F2 is also annotated as a TF of ITGA6, with neighboring binding sites to YY1 (Figure 5C). A correlation analysis between human annotated TFs (*n* = 1508) and YY1 expression in TCGA and Ital–Mex (GSE86948) cohorts described a set of TFs significantly co-expressed with YY1 including: ATF1, ATF2, CREB, E2F2, E2F3, and SP3 (Figure S6). Most of these TFs present a similar relevance score (above 3rd quartile) that indicates the confidence of the predicted interaction based on experimental evidence and database information (STRING [32]) reported by GeneCards [33] (Figure 5D). According to this information, we speculated that when miR-302b is re-introduced, E2F2 is not affected and is still expressed; therefore, it is able to interact with YY1 contributing to activate ITGA6 transcription. Indeed, only concomitant treatment with miR-302b and cisplatin significantly downmodulated E2F2, YY1, and ITGA6 (Figure 6), promoting TNBC cisplatin sensitivity.

Taken together, these results suggest that the combinatorial effect of miR-302b and cisplatin on TNBC cell viability is exerted thought a mechanism involving the E2F family, YY1 and ITGA6, all molecules modulated by cisplatin activity and implicated in the response to this drug.

### 2.4. miR-302b as a Prognostic Factor in TNBC

**Figure 7:**
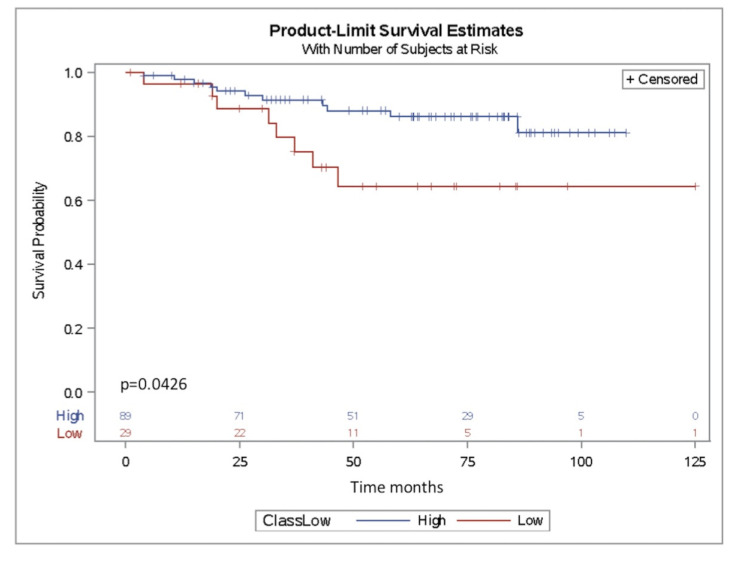
miR-302b as prognostic factor in TNBC patients. Kaplan–Meier survival curves of patients stratified according to miR-302b expression. Patients were dichotomized using first quartile as the threshold. Curves represent overall survival based on miR-302b expression (*p* ≤ 0.05, log-rank test).

To evaluate the prognostic impact of miR-302b, we analyzed the association of its expression level with overall survival in our cohort of TNBC (Ital–Mex) patients treated with adjuvant chemotherapy and previously profiled for miRNA expression (*n* = 118) (GSE86948). Patients were then grouped according to miR-302b expression as follows: low expression (below 1st quartile) or high expression (above 1st quartile). Kaplan–Meier analysis showed that patients whose tumors had lower miR-302b also had shorter overall survival (*p* = 0.0426) (Figure 7). Taken together, these results suggest that miR-302b may be used as a prognostic biomarker in TNBC patients.

## 3. Discussion

In this study, we demonstrated that miR-302b could serve as a tool to enhance the sensitivity to cisplatin chemotherapy, through the modulation of a complex biological network, never investigated before, that promotes response to the chemotherapeutic compound. Starting from our previous evidence indicating that miR-302b is able to improve sensitivity to cisplatin in breast cancer in vitro models [12], we treated TNBC xenografted mice with miR-302b mimic or cel-miR-67 negative control, in combination or not with cisplatin. Our data demonstrated that only concomitant treatment with the miR-302b mimic and cisplatin reduced tumor growth. Primary and/or acquired resistance to therapies is a major hurdle in clinical oncology, and it raises two main needs: the definition of biomarkers useful to select patients who will actually benefit from a specific treatment and the need to develop novel efficient therapeutic approaches. Our result represents a proof of concept for a future miR-302b-based therapy for TNBC patients. In the era of molecular and personalized therapy, cytotoxic chemotherapy is still the standard-of-care as a first line treatment for TNBC patients. Moreover, patients often present intrinsic resistance to the treatment and will eventually become non-responsive with an impaired clinical benefit. MicroRNAs have been reported to be strongly involved in therapy response in cancer models including TNBC. MicroRNAs in cancer therapy have been highly discussed and one of the main concerns when proposing miRNA-based therapy is the feasibility of an approach that has not always been successful in the past (e.g., miRX34 trial (miR-34a mimics) has failed due to the fact of its severe toxicity) However, there are also encouraging examples such as Mesomir, a miR-16 mimic which has successfully completed phase II, and a couple of years ago, the LNA-modified anti-miR-155 was tested in a phase I clinical trial in patients with cutaneous T-cell lymphoma. These on-going clinical trials taking advantage of miRNA inhibitors or mimics are encouraging examples that a miRNA-based therapy might represent a feasible a promising novel approach. Since one of the major concerns is the miRNA potential off-target effects, our long-term goal is to develop a strategy to deliver miR-302b with a tumor-specific method to overcome the issues of potential side effects.

We also aimed at understanding the mechanism behind the miR-302b and cisplatin combination. First, we tested the expression of E2F1, a known target gene of miRNA based on our previous data in tumor mice treated with miR-302b mimics and negative control with or without cisplatin. Surprisingly, the expression of E2F1 was not reduced in mice treated with cisplatin (data not shown). This phenomenon can be explained through different biological settings. For instance, miRNA and gene expression analysis of in vivo experiment was evaluated only at a final time-point; thus, direct miR-302b activity might be an early regulation process that occurs in the first tumorigenic steps. Instead, down-stream indirect pathways mediated by miR-302b might be altered as a late molecular mechanism and cooperate with other processes in vivo to attenuate oncogenic features as we described in this study. In addition to this, it is also conceivable that residual tumoral tissue escaped E2F1 downregulation mediated by miR-302b or rescued its expression, being E2F1 crucial for cell proliferation. Analyzing the transcriptional landscape upon miR-302b and cisplatin combined treatment in vivo, we demonstrated ITGA6 down-modulation. ITGA6 belongs to Integrin family, it is a transmembrane glycoprotein adhesion receptor involved in cell–matrix and cell–cell adhesion. It is overexpressed in breast cancer and associated with poor prognosis [34]. Notably, ITGA6 is a cancer stem cell marker, also known as CD49f [35]. Recently, it has been demonstrated that ITGA6 mediates radio- and chemo-resistance in breast cancer. Specifically, CD49f-positive cells with tumor-initiating capability are present in chemoresistant population in responsive TNBC model, and this population expands when tumors acquire resistance [24]. Moreover, consistent with our previous data concerning the capability of miR-302b to affect DNA repair, ITGA6 has been associated to a reduced sensitivity to radiotherapy by improving the capability to repair DNA damage [34]. This information led us to focus our attention on this gene. Phenocopy experiments performed transfecting si-ITGA6 in two TNBC cell lines demonstrated that ITGA6 downregulation had the same effect as miR-302b reintroduction on cisplatin sensitivity. Despite ITGA6 silencing at least partially recapitulating an miR-302b-triggered phenotype, it is conceivable that miR-302b’s biological effects are mediated by regulation of additional direct and/or indirect targets.

Consistently, in silico analyses of public data sets on TNBC patients, treated with neoadjuvant cisplatin chemotherapy, revealed that patients with partial response or progressive diseases have high levels of ITGA6. However, ITGA6 is not a predicted target of miR-302b; thus, we explored possible cooperative regulatory networks of ITGA6, such as TFs, that might be altered by miR-302b and cisplatin intervention. Our results suggest a regulatory mechanism involving the E2F family and YY1. Relevantly, the components of the biological axis E2F/ITAG6/YY1 were significantly up-modulated in TNBC tumors in comparison with other tumor types (Figure S7), corroborating our hypothesis that miR-302b reintroduction would have a major effect on this subtype.

Yin Yang 1 is a transcription factor, highly conserved and ubiquitously expressed, which can activate or repress diverse cancer-related targets [36], such as genes involved in cell proliferation and differentiation, DNA repair, chromatin modeling, apoptosis, and contributes to the aberrant epigenetic mechanisms of cancers. However, even though many studies report YY1 as an oncogenic protein in many cancers, in breast cancer it has a controversial role as an oncogene or oncosuppressor [36]. Some studies revealed that YY1 is overexpressed in breast cancer [37], correlating with poor prognosis [38]. Moreover, several studies have demonstrated that YY1 is involved in cisplatin responsiveness, in different cancer models. For example, Zhao et al. [39] have recently demonstrated that knock-down of YY1 promotes cisplatin anticancer effects in head and neck squamous cell carcinoma. Schlisio et al. [31] have also shown that YY1 binds to other relevant transcription factors, such as E2F2 and E2F3, but not with E2F1, to promote Cdc6 transcription which has been linked to human cancer development. The E2F family has a key role in cell cycle progression; it is divided into four groups based on their function [40]. Particularly, E2Fs 1–3 are important for the correct progression through cell cycle, and their loss induces cell cycle arrest and, consequently, cell death. Although E2F1 has both an oncogenic and oncosuppressive role [41], it was found that E2F1 overexpression is correlated to poor prognosis in breast cancer [42]. Loss of E2F2 and E2F3 is related to a significant delay in tumor onset. E2F transcription factor 2 loss led to downregulation of the EMT process. In human breast cancer samples, low activation of E2F2 is associated with increased relapse-free survival time [41]. These data were consistent with our hypothetical molecular mechanism behind the acquired chemo-sensitivity of TNBC cells mediated by miR-302b. In particular, whereas miR-302b directly targets E2F1, the redundancy of other E2F family member functions is likely to maintain downstream molecules as YY1 and ITGA6 expression when cell faces miR-302b monotreatment. The existence of redundant and compensatory mechanisms might also explain why miR-302b alone is not able to significantly affect tumor growth. Only the combined treatment of miR-302b plus cisplatin leads to a significant down-modulation of E2F1, E2F2, and YY1 which negatively affect ITGA6 transcription and protein level. Thus, this suggests a cooperation between the cellular impact of cytotoxic cisplatin treatment and the molecular regulatory mechanism of miR-302b. However, the exact mechanism behind this synergy still needs to be demonstrated.

The evidence that YY1 is able to physically interact with E2F2 and E2F3 but not E2F1 [31] must be addressed in future experiments to confirm the physical binding between E2F2–YY1 in the ITGA6 promoter sequence and the relevance of this protein–protein interaction in our biological phenomena. Conversely, diverse evidence shows a clear association between loss or inactivation of tumor-suppressor genes that result in tumor development. In our combined cohort of TNBC patients treated with adjuvant chemotherapy from Fundación para Enfermedades de la Mama FUCAM (Mexico) and from Fondazione IRCCS Istituto Nazionale dei Tumori di Milano (Italy), miR-302b was detected at very low levels as expected by its oncosuppressive role. For instance, in other available public cohorts, such as The Cancer Genome Atlas (TCGA) and Molecular Taxonomy of Breast Cancer International Consortium (METABRIC), miR-302b was undetectable, probably by the features and coverage power of the employed technologies. Setting aside this, we were able to describe an association between increased miR-302b expression and a better overall survival as a significant prognostic factor in TNBC.

Thus, our published results [12] and this present work confirm that miR-302b affects different molecular pathways, such as DNA repair, cell cycle and stemness, and enhances the response to cisplatin. Overall, miR-302b is a novel candidate as therapeutic tool able to enhance the response to cisplatin in TNBC through the modulation of E2F family–YY1–ITGA6 axis. Finally, our results demonstrated that miR-302b has also a prognostic value in TNBC patients.

## 4. Materials and Methods

### 4.1. Cell Lines

Human TNBC cell lines MDA-MB-231 and BT549 were purchased from ATCC. They were authenticated annually (last verification on November 2018) using the short tandem repeat profiling method and propagated within 6 months of thawing from stocks. Cells were maintained in RPMI 1610 medium with 10% FBS and 1 mmol/L L-glutamine, at 37 °C in a humidified atmosphere of 5% CO^2^ in air. The MycoAlert Mycoplasma Detection Kit (Lonza, Basel, Switzerland) was used to assure a negative mycoplasma status in cultured cells before experiments were started.

### 4.2. In Vivo Experiment

In vivo experiments were performed with 8 week old female immunodeficient Fox Chase SCID mice (CB17/Icr-Prkdcscid/IcrIcoCrl) purchased from Charles Rivers. Mice were housed at the animal facility of Fondazione IRCCS Istituto Nazionale dei Tumori of Milan. All animal experiments were approved by the Ethics Committee for Animal Experimentation of Fondazione IRCSS Istituto Nazionale dei Tumori of Milan.

For treatments, we used miR-302b mimic or cel-miR-67 negative control (Tema Ricerca, Castenaso (BO), Italy). Dried miRNA oligonucleotides were resuspended in sterile water to obtain a 10 mg/mL stock. The MiRNAs were then formulated within lipid nanoparticles using MaxSuppressor In Vivo RNA-LANCEr II (Bioo Scientific, Austin, TX, USA). Cisplatin was obtained from Fondazione IRCCS Istituto Nazionale dei Tumori pharmacy and diluted in physiological saline at 1 mg/mL concentration.

Orthotopic triple-negative breast tumors were generated in mice (*n* = 28) by injecting 5 × 10^6^ cells/each of MDA-MB-231 cells, resuspended in 200 µL of a 1:1 mixture with PBS and Matrigel (Corning, New York, NY, USA), in the mammary fat pad. When tumors were palpable (50 mm^3^), mice were divided into 4 groups of 7 mice each, maintaining the same mean tumor volume in all groups. Two groups of mice were treated with 50 µL of lipid nanoparticles containing 20 µg miR-302b mimic and the other 2 groups with 50 µL of lipid nanoparticles containing 20 µg cel-miR- 67 negative control administered every 3 days by peritumoral injection. After 24 h of each nanoparticle treatment, both groups of mice treated with miR-302b mimic and the one treated with cel-miR- 67 negative control received an intraperitoneal injection of 2 mg/kg cisplatin, while the others were treated with an equal volume of physiological saline solution. Tumor growth was measured every 3 days using a caliper, and at the end of the five treatment cycles, mice were euthanized. Finally, tumors were collected and frozen at −80 °C.

### 4.3. mRNA Expression Profiling of In-Vivo Model Tumor Tissue

Twenty-four collected mice tumors were homogenized, and total RNA was isolated with Trizol reagent (Thermo Fischer Scientific, Waltham, MA, USA) by the Functional Genomics and Bioinformatics Core facility of Fondazione IRCCS Istituto Nazionale dei Tumori of Milan. RNA quality was assessed with the Agilent Bioanalyzer (Agilent Technologies, Santa Clara, CA, USA) using the RNA 6000 Nano Kit (Agilent Thecnologies, Santa Clara, CA, USA). RiboNucleic Acid (RNA) concentrations were measured with the NanoDrop ND-100 spectrophotometer (NanoDrop Technologies, Wilmington, DE, USA). Gene profiling analysis was performed with HumanHT-12 V4 expression Chip (Illumina, Inc., San Diego, CA, USA). Briefly, 800 ng of total RNA was reverse transcribed, biotin labeled, amplified, and hybridized as indicated by the manufacturer’s instructions. Array chips were then scanned by an Illumina BeadArrayTM reader in the Functional Genomics and Bioinformatics Core facility.

### 4.4. Gene Expression Analysis

Raw expression data were processed using the lumi package [43] on Bioconductor. Gene raw data were normalized with quantile algorithm and log2transformed. Probes with a detection *p* < 0.01 were maintained for the analysis. Quality control was performed on raw and processed data, all samples fulfilling quality control procedures were included (*n* = 23). Probes were annotated using the illuminaHumanv4.db package [44] and biomart [45] R dependency. Genes evaluated by multiple probes were collapsed by the highest detection value. Differentially expressed genes among the biological conditions (treated with: miR-302b (*n* = 5), miR-302b+cisplatin (*n* = 6), cel-miR-67 control (*n* = 6), and cel-miR-67 control+cisplatin (*n* = 6)) were identified by linear modeling implemented in limma package [46] of Biocondutor on R environment. Genes with a log fold change of ≥0.5 or ≤−0.5 and *p*-value < 0.05 were considered as significantly differentially expressed. Expression data were deposited in the repository of functional genomics data from array- and sequence-based analyses (GEO) with accession number GSE152322.

### 4.5. Pathway Analysis

Comprehensive pathway enrichment analysis was performed with the InnateDB (https://www.innatedb.com/) [47] and David (https://david.ncifcrf.gov/content.jsp?file=Acknowledgement.htm [48]) databases using Kegg, Reactome, and biological process gene ontology as annotations. Pathways with adjusted, and *p*-values ≤ 0.05 were considered as significant enriched signaling.

### 4.6. Independent In Silico Analysis of Cisplatin Mediated Genes and its Pathological Response Association

Normalized gene expression data from the GEO repository was evaluated as independent datasets to define the correlation between ITGA6 expression and clinical cisplatin treatment response. First dataset (GSE18864) studied TNBC patients treated with a single arm neoadjuvant trial with cisplatin (*n* = 24), and the second one (GSE103668) evaluated 21 TNBC samples treated with cisplatin and bevacizumab in neoadjuvant; for this analysis, we only included those tumors treated only with cisplatin (*n* = 17).

### 4.7. Transcription Factor Motif Analysis by Benchmark Databases

To identify the transcription factors predicted to be directly involved on transcriptomic changes, we applied two position weight matrices (PWMs) enrichment methods as a predictive model to identify putative TFs based on the differentially expressed genes resulted from the in vivo experiment (mice treated with miR-302b+cisplatin versus mice treated with miR-302b alone): (1) oPOSSUM on-line tool [25], that computes two scoring methods to measure the over-representation of transcription factor binding sites: a z-scores that measures the change in the relative number of TFBS motifs in the differentially expressed genes compared with the background set, and one-tailed Fisher exact probability assessing the number of genes with the TFBS motifs in the cutting-edge set versus the background set; and (2) PASTAA (Predict Associated Transcription Factors from Annotated Affinities) [26,27] that uses binding affinities of TFs to detect binding motifs. All TFs were ranked according to their predicted affinity for the given down-expressed genes. All analyses were run with default parameters. Only those predicted TFs found in both analyses were further considered. TF::miRNA interactions were inferred with mirWalk v2 (http://zmf.umm.uni-heidelberg.de/apps/zmf/mirwalk2/index.html) [49] with the following algorithms: miRWalk, Microt4, miRanda, mirbridge, miRDB, miRMap, miRNAMap, Pictar2, PITA, RNA22, RNAhybrid, and Targetscan. Thus, only predicted TFs annotated by 3 or more algorithms were retained.

Then, the JASPAR 2020 Core vertebrate database (Human) (http://jaspar.genereg.net/) [28,29] was used as the source of DNA binding motifs of YY1, E2F1, IRF1, FOXO3, and SP1 to explore ITGA6 promoter sequence (−3000 bp from TSS). All binding motifs were scanned with JASPAR to predict binding sites. Threshold to determine the degree of potential binding sites occurrence were defined as a relative profile score threshold > 0.9. The analysis was visualized in R using ggplot. Next, ChIP-Atlas (https://chip-atlas.org/) [30], a data-mining suite to explore peak-call for public ChIP-seq data, was applied. The TF binding sites were filtered for those with a MACS peak Q-value > 200 and from all human available cell lines and tissues. The TFs were accepted if their peak-call overlapped with the ITGA6 promoter sequence (−3000 pb from TSSS). Peaks and coverage were visualized with IGV [50,51] (https://software.broadinstitute.org/software/igv/). The transcription factor binding site hits were evaluated as following: the proportion of true hits (those motifs that fall within down-modulated genes by miR-302b+cisplatin treatment) versus false hits (those motifs that fall within genes which have not been altered by miR-302b+cisplatin treatment background genes). The YY1 protein interaction was calculated with information from GeneCards suite [33] (https://www.genecards.org/) and Opossum/PASTAA results.

### 4.8. Data Mining of TNBC Patients from TCGA and ITAL–MEX Data Sets

Transcriptome data from TNBC analyzed on The Cancer Genome Atlas (TCGA, *n* = 162) were downloaded from the Xena browser (UCSC Xena http://xena.ucsc.edu) [52] the GDC TCGA Breast Cancer cohort (HTSeq—counts of gene expression RNAseq). Low normalized counts (<10) of data were filtered. Normalization procedures were computed using DESeq [53] in an R environment. Normalized mRNA expression data from Ital–Mex cohort (*n* = 158 TNBC) were obtained from GEO Omnibus database with accession number GSE86948. Spearman correlation analysis between gene expression profiles of human TFs (*n* = 1508), retrieved from TcoF-DB v2 database (https://tools.sschmeier.com/tcof/home/) [54], and YY1 expression level was performed. Only pairs with a correlation coefficient >30% and Benjamini–Hochberg adjusted *p*-value < 0.05 were considered significant.

### 4.9. miRNA and siRNA Transfection

MiRNA overexpression was achieved by transfection with human miR-302b precursor or negative control (Thermo Fisher Scientific) and verified by qRT-PCR. For gene knockdown, specific siRNAs, si-ITGA6, si-E2F1, or their negative control (Thermo Fisher Scientific) were used. Cells were transfected with 100 nmol/L miRNA precursor or siRNAs and their negative controls complexed with Lipofectamine 2000 reagent (Thermo Fisher Scientific) according to the manufacturer’s instructions.

### 4.10. Quantitative RT-PCR

Total RNA from mice tumors was used to synthesize cDNA with TaqMan MiRNA Reverse Transcription Kit (Thermo Fischer Scientific). The qRT-PCR was then performed using TaqMan assays for miR-302b and small-nucleolar RNA RNU44, E2F1, and GAPDH (Thermo Fischer Scientific). The MiR-302b and E2F1 levels in the mice tumors were normalized to the endogenous control RNU44 and GAPDH, respectively, and the relative expression was calculated using the comparative 2^−ΔCt^ method.

### 4.11. Western Blot Analysis

Total protein lysates were extracted with lysis buffer (1% Triton, 50 nmol/L Tris, 15 mmol/L NaCl) supplemented with protease inhibitors (Sigma–Aldrich, St. Louis, MO, USA). The following primary antibodies were used:: ITGA6 (recombinant rabbit monoclonal antibody, clone: EPR18124, catalog number: ab181551, Abcam), E2F1 (mouse polyclonal antibody, clone: C-20, catalog number: sc-193, Santa Cruz), YY1 (mouse monoclonal antibody, clone: 2E11C5, catalog number: 66281-1-Ig, Proteintech, Manchester, United Kingdom), E2F2 (mouse monoclonal antibody, clone: TFE-25, catalog number: sc-9967, Santa Cruz Biothecnology, Dallas, TX, USA), β-actin (mouse monoclonal Anti-β-Actin−Peroxidase antibody, clone: AC-15, catalog number: A3854, Sigma–Aldrich, St. Louis, MO, USA) and vinculin (mouse monoclonal antibody, clone: hVIN-1, catalog number: V9131, Sigma–Aldrich, St. Louis, MO, USA). Proteins were visualized by enhanced chemiluminescence detection system (Sigma–Aldrich, St. Louis, MO, USA). Quantification was performed by Quantity One 4.6.6 software (Bio-Rad, Hercules, California, USA).

### 4.12. miRNA Expression Profiling from TNBC Patients

The miRNA expression profiles from 132 triple-negative breast cancer tumors were retrieved from our super series GSE86948 of GEO omnibus database. Overall survival information was available from 118 tumors. Patients were divided into miR-302b low set (below 1st quartile) and high set (above 1st quartile) for clinical statistical analysis.

### 4.13. Statistical Analysis

The statistical significance of the in vivo experiment was calculated with unpaired two-tailed Student’s *t*-test. Survival curves were generated using the Kaplan–Meier method and tested with log-rank test, using SAS software (SAS Institute, Cary, NC, USA). *p* ≤ 0.05 was considered significant.

## 5. Conclusions

Our study demonstrated that miR-302b improves the sensitivity to cisplatin in TNBC models in vitro and in vivo. The efficacy of miR-302b and cisplatin combination is mediated by the inhibition of the E2F family and YY1 TFs with consequent impairment of ITGA6 activation and improved cisplatin sensitivity. Our data represent the proof of concept for a future use of miR-302b as miRNA-based therapy in combination with cisplatin for TNBC patients. Finally, the correlation of miR-302b expression with better overall survival in our Ital–Mex cohort indicates that miR-302b might be used as a prognostic biomarker in TNBC.

## Supplementary Materials

The following are available online at https://www.mdpi.com/2072-6694/12/8/2261/s1: Figure S1. Relative miRNA-302b levels in the four in vivo groups. Data are presented as mean and SD of all tumors from each mice group. Figure S2. Western Blot analysis of Integrin Subunit Alpha 6 (ITGA6) protein expression in mice tumors treated with miR-302b mimic alone and cel-miR-67 alone. Upper figure shows specific ITGA6 bands and vinculin bands as housekeeping; lower histogram represents ITGA6 densitometric quantification. Figure S3. BT549 cell count after small interfering-ITGA6 (si-ITGA6) transfection and cisplatin treatment compared with their negative controls. Data are representative of three independent experiments performed at least by triplicate (* *p* ≤ 0.05 Student’s *t* test). Figure S4. Veen diagram showing the overlap of enriched Transcription Factors (TFs) whose binding sites are overrepresented in the promoter of the differentially expressed genes in in vivo assay (miR-302b+cisplatin *versus* cel-miR-67 negative control+cisplatin). Identification of enriched transcription factors using oPOSSUM and PASTAA algorithms. Figure S5. Densitometric protein quantifications of figures 4 and 5, performed with QuantiyOne tool. Figure S6. Correlation matrix of Yin Yang 1 (YY1) and human TFs in TNBC cases of ITAL-MEX and TCGA cohorts computed by Spearman correlation. Purple areas indicate strong positive correlation. Only genes with a significant correlation (BH adjusted p< 0.05) were colored. Figure S7. Normalized expression profiles of E2F/ITAG6/YY1 *axis on* TCGA and METABRIC cohorts. Table S1: Gene expresion profiles of in-vivo assay evalauted by microarray analysis. Table S2: Summary of the TFs in silico analysis including oPOSSUM, PASTAA and miRwalk results. Table S3: Summary of in-silico JASPAR analysis of the selected TFs.

## References

[1] Iorio M.V., Croce C.M. (2012). MicroRNA dysregulation in cancer: Diagnostics, monitoring and therapeutics. A comprehensive review. EMBO Mol. Med..

[2] Betel D., Wilson M., Gabow A., Marks D.S., Sander C. (2008). The microRNA.org resource: Targets and expression. Nucleic Acids Res..

[3] Ling H., Fabbri M., Calin G.A. (2013). MicroRNAs and other non-coding RNAs as targets for anticancer drug development. Nat. Rev. Drug Discov..

[4] Garofalo M., Croce C.M. (2013). MicroRNAs as therapeutic targets in chemoresistance. Drug Resist. Updat..

[5] Raguz S., Yague E. (2008). Resistance to chemotherapy: New treatments and novel insights into an old problem. Br. J. Cancer..

[6] Symmans W.F., Wei C., Gould R., Yu X., Zhang Y., Liu M., Walls A., Bousamra A., Ramineni M., Sinn B. (2017). Long-Term Prognostic Risk After Neoadjuvant Chemotherapy Associated With Residual Cancer Burden and Breast Cancer Subtype. J. Clin. Oncol..

[7] Lebert J.M., Lester R., Powell E., Seal M., McCarthy J. (2018). Advances in the systemic treatment of triple-negative breast cancer. Curr. Oncol..

[8] Peto R., Davies C., Godwin J., Gray R., Pan H.C., Clarke M., Cutter D., Darby S., McGale P., Taylor C. (2012). Comparisons between different polychemotherapy regimens for early breast cancer: Meta-analyses of long-term outcome among 100,000 women in 123 randomised trials. Lancet.

[9] Foulkes W.D., Smith I.E., Reis-Filho J.S. (2010). Triple-negative breast cancer. N. Engl. J. Med..

[10] Crown J., O’Shaughnessy J., Gullo G. (2012). Emerging targeted therapies in triple-negative breast cancer. Ann. Oncol..

[11] Kelland L.R. (2000). Preclinical perspectives on platinum resistance. Drugs.

[12] Cataldo A., Cheung D.G., Balsari A., Tagliabue E., Coppola V., Iorio M.V., Palmieri D., Croce C.M. (2016). miR-302b enhances breast cancer cell sensitivity to cisplatin by regulating E2F1 and the cellular DNA damage response. Oncotarget.

[13] Suh M.R., Lee Y., Kim J.Y., Kim S.K., Moon S.H., Lee J.Y., Cha K.Y., Chung H.M., Yoon H.S., Moon S.Y. (2004). Human embryonic stem cells express a unique set of microRNAs. Dev. Biol..

[14] Ren J., Jin P., Wang E., Marincola F.M., Stroncek D.F. (2009). MicroRNA and gene expression patterns in the differentiation of human embryonic stem cells. J. Transl. Med..

[15] Chen P.H., Shih C.M., Chang W.C., Cheng C.H., Lin C.W., Ho K.H., Su P.C., Chen K.C. (2014). MicroRNA-302b-inhibited E2F3 transcription factor is related to all trans retinoic acid-induced glioma cell apoptosis. J. Neurochem..

[16] De Cecco L., Berardi M., Sommariva M., Cataldo A., Canevari S., Mezzanzanica D., Iorio M.V., Tagliabue E., Balsari A. (2013). Increased sensitivity to chemotherapy induced by CpG-ODN treatment is mediated by microRNA modulation. PLoS ONE.

[17] Wang L., Yao J., Zhang X., Guo B., Le X., Cubberly M., Li Z., Nan K., Song T., Huang C. (2014). miRNA-302b suppresses human hepatocellular carcinoma by targeting AKT2. Mol. Cancer Res..

[18] Zhang M., Yang Q., Zhang L., Zhou S., Ye W., Yao Q., Li Z., Huang C., Wen Q., Wang J. (2014). miR-302b is a potential molecular marker of esophageal squamous cell carcinoma and functions as a tumor suppressor by targeting ErbB4. J. Exp. Clin. Cancer Res..

[19] Zhu R., Yang Y., Tian Y., Bai J., Zhang X., Li X., Peng Z., He Y., Chen L., Pan Q. (2012). Ascl2 knockdown results in tumor growth arrest by miRNA-302b-related inhibition of colon cancer progenitor cells. PLoS ONE.

[20] Liang Z., Ahn J., Guo D., Votaw J.R., Shim H. (2013). MicroRNA-302 replacement therapy sensitizes breast cancer cells to ionizing radiation. Pharm. Res..

[21] Wang Y., Zhao L., Xiao Q., Jiang L., He M., Bai X., Ma M., Jiao X., Wei M. (2016). miR-302a/b/c/d cooperatively inhibit BCRP expression to increase drug sensitivity in breast cancer cells. Gynecol. Oncol..

[22] Zhao L., Wang Y., Jiang L., He M., Bai X., Yu L., Wei M. (2016). MiR-302a/b/c/d cooperatively sensitizes breast cancer cells to adriamycin via suppressing P-glycoprotein(P-gp) by targeting MAP/ERK kinase kinase 1 (MEKK1). J. Exp. Clin. Cancer Res..

[23] Ma J., Zhou Z. (2020). Downregulation of miR-302b is associated with poor prognosis and tumor progression of breast cancer. Breast Cancer.

[24] Gomez-Miragaya J., Gonzalez-Suarez E. (2017). Tumor-initiating CD49f cells are a hallmark of chemoresistant triple negative breast cancer. Mol. Cell Oncol..

[25] Kwon A.T., Arenillas D.J., Worsley Hunt R., Wasserman W.W. (2012). oPOSSUM-3: Advanced analysis of regulatory motif over-representation across genes or ChIP-Seq datasets. G3 (Bethesda).

[26] Roider H.G., Kanhere A., Manke T., Vingron M. (2007). Predicting transcription factor affinities to DNA from a biophysical model. Bioinformatics.

[27] Roider H.G., Manke T., O’Keeffe S., Vingron M., Haas S.A. (2009). PASTAA: Identifying transcription factors associated with sets of co-regulated genes. Bioinformatics.

[28] Stormo G.D. (2013). Modeling the specificity of protein-DNA interactions. Quant. Biol..

[29] Wasserman W.W., Sandelin A. (2004). Applied bioinformatics for the identification of regulatory elements. Nat. Rev. Genet..

[30] Oki S., Ohta T., Shioi G., Hatanaka H., Ogasawara O., Okuda Y., Kawaji H., Nakaki R., Sese J., Meno C. (2018). ChIP-Atlas: A data-mining suite powered by full integration of public ChIP-seq data. EMBO Rep..

[31] Schlisio S., Halperin T., Vidal M., Nevins J.R. (2002). Interaction of YY1 with E2Fs, mediated by RYBP, provides a mechanism for specificity of E2F function. EMBO J..

[32] Szklarczyk D., Gable A.L., Lyon D., Junge A., Wyder S., Huerta-Cepas J., Simonovic M., Doncheva N.T., Morris J.H., Bork P. (2019). STRING v11: Protein-protein association networks with increased coverage, supporting functional discovery in genome-wide experimental datasets. Nucleic Acids Res..

[33] Stelzer G., Rosen N., Plaschkes I., Zimmerman S., Twik M., Fishilevich S., Stein T.I., Nudel R., Lieder I., Mazor Y. (2016). The GeneCards Suite: From Gene Data Mining to Disease Genome Sequence Analyses. Curr. Protoc. Bioinformatics.

[34] Hu T., Zhou R., Zhao Y., Wu G. (2016). Integrin alpha6/Akt/Erk signaling is essential for human breast cancer resistance to radiotherapy. Sci. Rep..

[35] Bigoni-Ordonez G.D., Czarnowski D., Parsons T., Madlambayan G.J., Villa-Diaz L.G. (2019). Integrin α6 (CD49f), The Microenvironment and Cancer Stem Cells. Curr. Stem Cell Res. Ther..

[36] Sarvagalla S., Kolapalli S.P., Vallabhapurapu S. (2019). The Two Sides of YY1 in Cancer: A Friend and a Foe. Front. Oncol..

[37] Thomassen M., Tan Q., A Kruse T. (2008). Gene expression meta-analysis identifies metastatic pathways and transcription factors in breast cancer. BMC Cancer.

[38] Qiao K., Ning S., Wan L., Wu H., Wang Q., Zhang X., Xu S., Pang D. (2019). LINC00673 is activated by YY1 and promotes the proliferation of breast cancer cells via the miR-515-5p/MARK4/Hippo signaling pathway. J. Exp. Clin. Cancer Res..

[39] Zhao L., Li R., Gan Y. (2018). Knockdown of Yin Yang 1 enhances anticancer effects of cisplatin through protein phosphatase 2A-mediated T308 dephosphorylation of AKT. Cell Death Dis..

[40] Attwooll C., Denchi E.L., Helin K. (2004). The E2F family: Specific functions and overlapping interests. EMBO J..

[41] Johnson J., Thijssen B., McDermott U., Garnett M., Wessels L.F., Bernards R. (2016). Targeting the RB-E2F pathway in breast cancer. Oncogene.

[42] Meng P., Ghosh R. (2014). Transcription addiction: Can we garner the Yin and Yang functions of E2F1 for cancer therapy?. Cell Death Dis..

[43] Smyth G.K. (2004). Linear Models and Empirical Bayes Methods for Assessing Differential Expression in Microarray Experiments. Stat. Appl. Genet. Mol. Boil..

[44] Dunning M., Lynch A., Eldridge M. (2015). Illuminahumanv4.db: Illumina HumanHT12v4 Annotation Data (Chip IlluminaHumanv4).

[45] Durinck S., Spellman P.T., Birney E., Huber W. (2009). Mapping identifiers for the integration of genomic datasets with the R/Bioconductor package biomaRt. Nat. Protoc..

[46] Ritchie M.E., Phipson B., Wu D., Hu Y., Law C.W., Shi W., Smyth G.K. (2015). limma powers differential expression analyses for RNA-sequencing and microarray studies. Nucleic Acids Res..

[47] https://www.innatedb.com/.

[48] Jiao X., Sherman B.T., Huang D.W., Stephens R., Baseler M.W., Lane H.C., Lempicki R.A. (2012). DAVID-WS: A stateful web service to facilitate gene/protein list analysis. Bioinformatics.

[49] Dweep H., Gretz N. (2015). miRWalk2.0: A comprehensive atlas of microRNA-target interactions. Nat. Methods.

[50] Robinson J.T., Thorvaldsdóttir H., Winckler W., Guttman M., Lander E.S., Getz G., Mesirov J.P. (2011). Integrative genomics viewer. Nat. Biotechnol..

[51] Thorvaldsdóttir H., Robinson J.T., Mesirov J.P. (2012). Integrative Genomics Viewer (IGV): High-performance genomics data visualization and exploration. Briefings Bioinform..

[52] http://xena.ucsc.edu.

[53] Love M.I., Huber W., Anders S. (2014). Moderated estimation of fold change and dispersion for RNA-seq data with DESeq2. Genome Biol..

[54] https://tools.sschmeier.com/tcof/home/.

