# Supplementary material for: MiR-302b as a Combinatorial Therapeutic Approach to Improve Cisplatin Chemotherapy Efficacy in Human Triple-Negative Breast Cancer
Source: Cancers (Basel). 2020 Aug 12;12(8):2261. doi: 10.3390/cancers12082261 (PMC7464985; doi:10.3390/cancers12082261)
Supplement: Supplementary file 1 [file cancers-12-02261-s001.zip › supplementary figure legends.docx]

**Figure S1.** Relative miRNA-302b levels in the four *in vivo* groups. Data are presented as mean and SD of all tumors from each mice group.

**Figure S2.** Western Blot analysis of Integrin Subunit Alpha 6 (ITGA6) protein expression in mice tumors treated with miR-302b mimic alone and cel-miR-67 alone. Upper figure shows specific ITGA6 bands and vinculin bands as housekeeping; lower histogram represents ITGA6 densitometric quantification.

**Figure S3.** BT549 cell count after small interfering-ITGA6 (si-ITGA6) transfection and cisplatin treatment compared with their negative controls. Data are representative of three independent experiments performed at least by triplicate (* p ≤ 0.05 Student’s *t* test).

**Figure S4.** Veen diagram showing the overlap of enriched Transcription Factors (TFs) whose binding sites are overrepresented in the promoter of the differentially expressed genes in *in vivo* assay (miR-302b+cisplatin *versus* cel-miR-67 negative control+cisplatin). Identification of enriched transcription factors using oPOSSUM and PASTAA algorithms.

**Figure S5**. Densitometric protein quantifications of figure 7 performed with QuantiyOne tool.

**Figure S6.** Correlation matrix of Yin Yang 1 (YY1) and human TFs in TNBC cases of ITAL-MEX and TCGA cohorts computed by Spearman correlation. Purple areas indicate strong positive correlation. Only genes with a significant correlation (BH adjusted p< 0.05) were colored.

**Figure S7.** Normalized expression profiles of E2F/ITAG6/YY1 *axis on* TCGA and METABRIC cohorts.

**Table S1:** Gene expresion profiles of in-vivo assay evalauted by microarray analysis

**Table S2:** Summary of the TFs in silico analysis including oPOSSUM, PASTAA and miRwalk results

**Table S3:** Summary of in-silico JASPAR analysis of the selected TFs.
