# Supplementary figures and images for: MiR-302b as a Combinatorial Therapeutic Approach to Improve Cisplatin Chemotherapy Efficacy in Human Triple-Negative Breast Cancer
Source: Cancers (Basel). 2020 Aug 12;12(8):2261. doi: 10.3390/cancers12082261 (PMC7464985; doi:10.3390/cancers12082261)

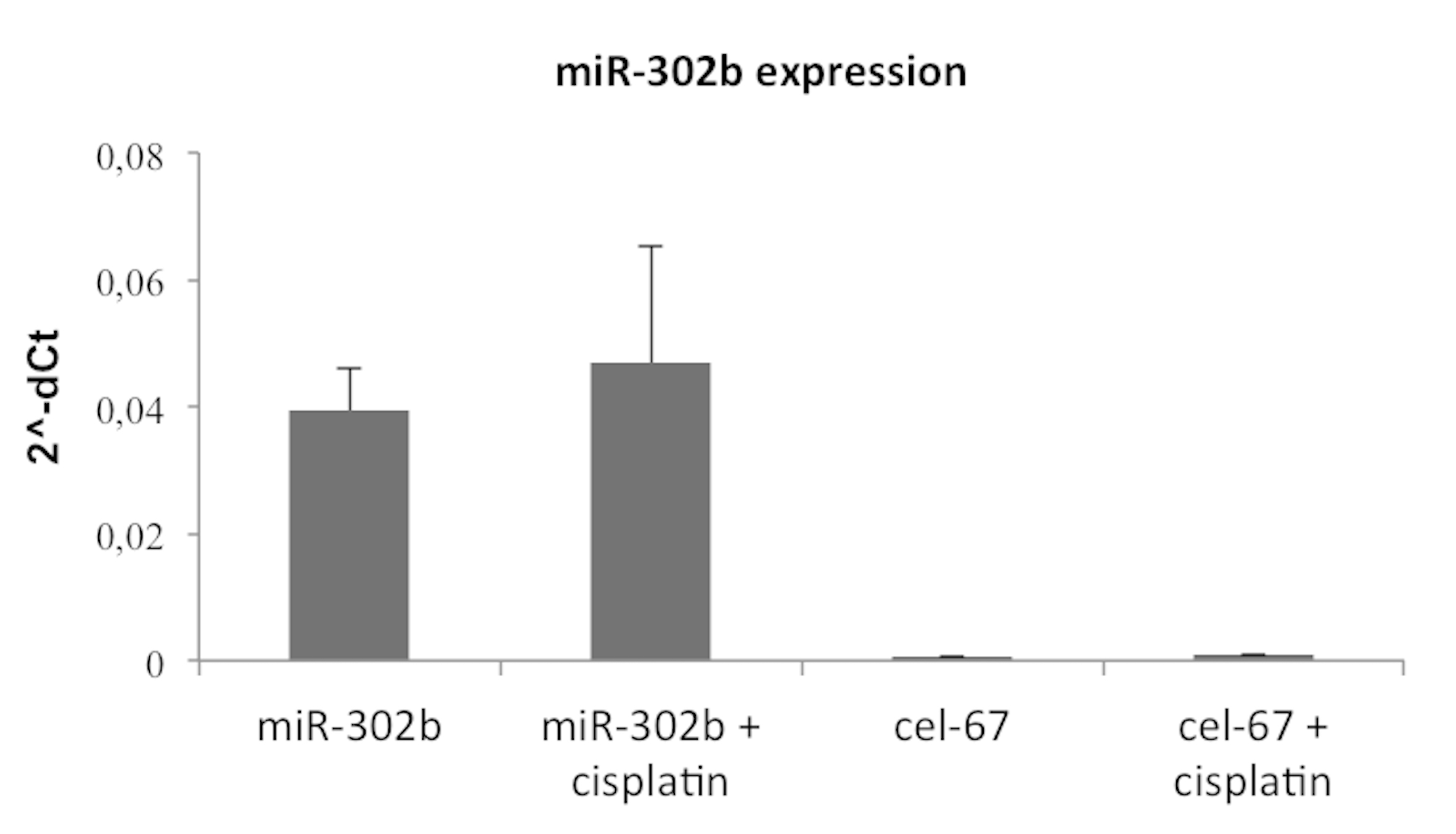

Supplement: Supplementary file 1 [file cancers-12-02261-s001.zip › Supplementary Figure 1.jpg]

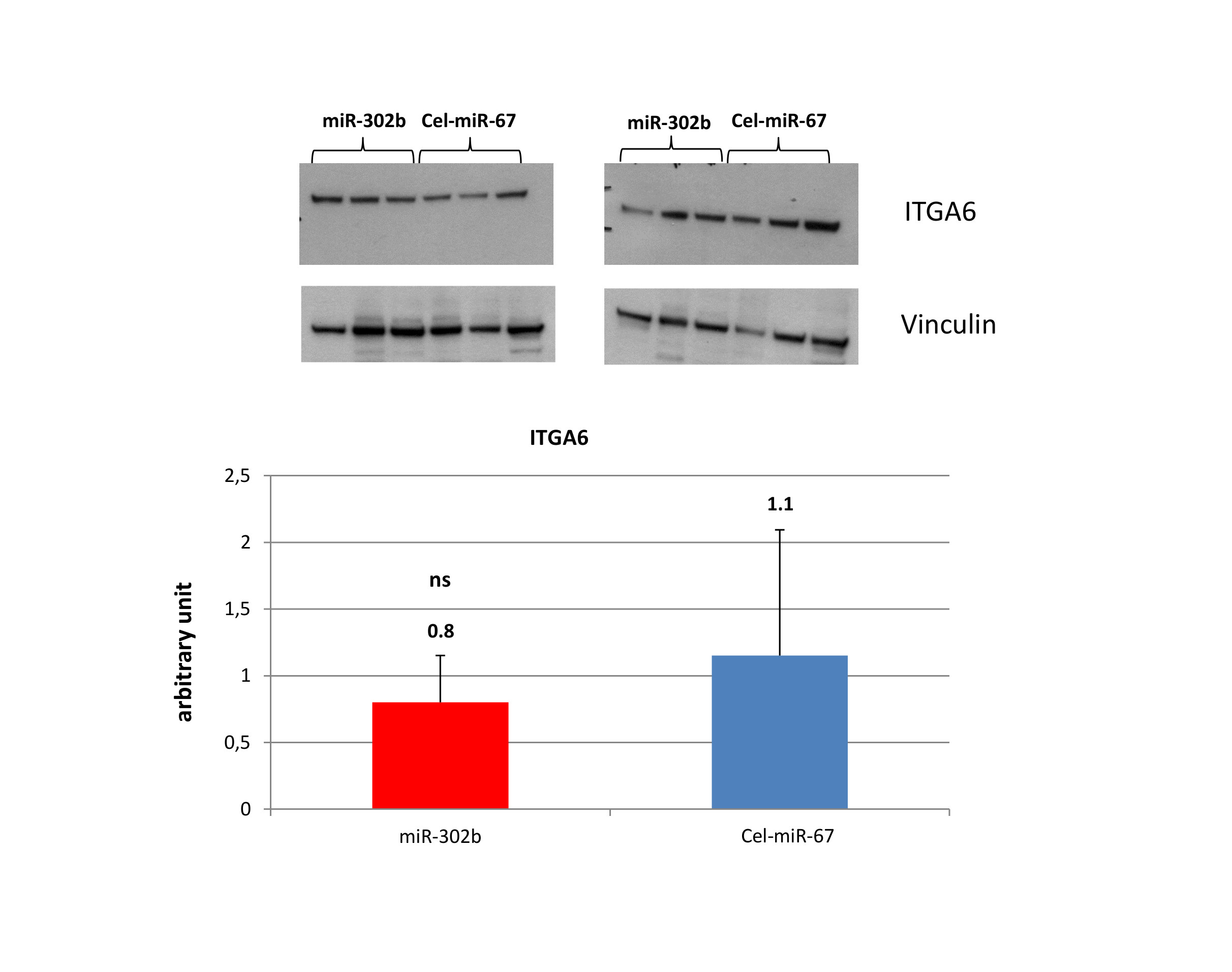

Supplement: Supplementary file 1 [file cancers-12-02261-s001.zip › Supplementary Figure 2.jpeg]

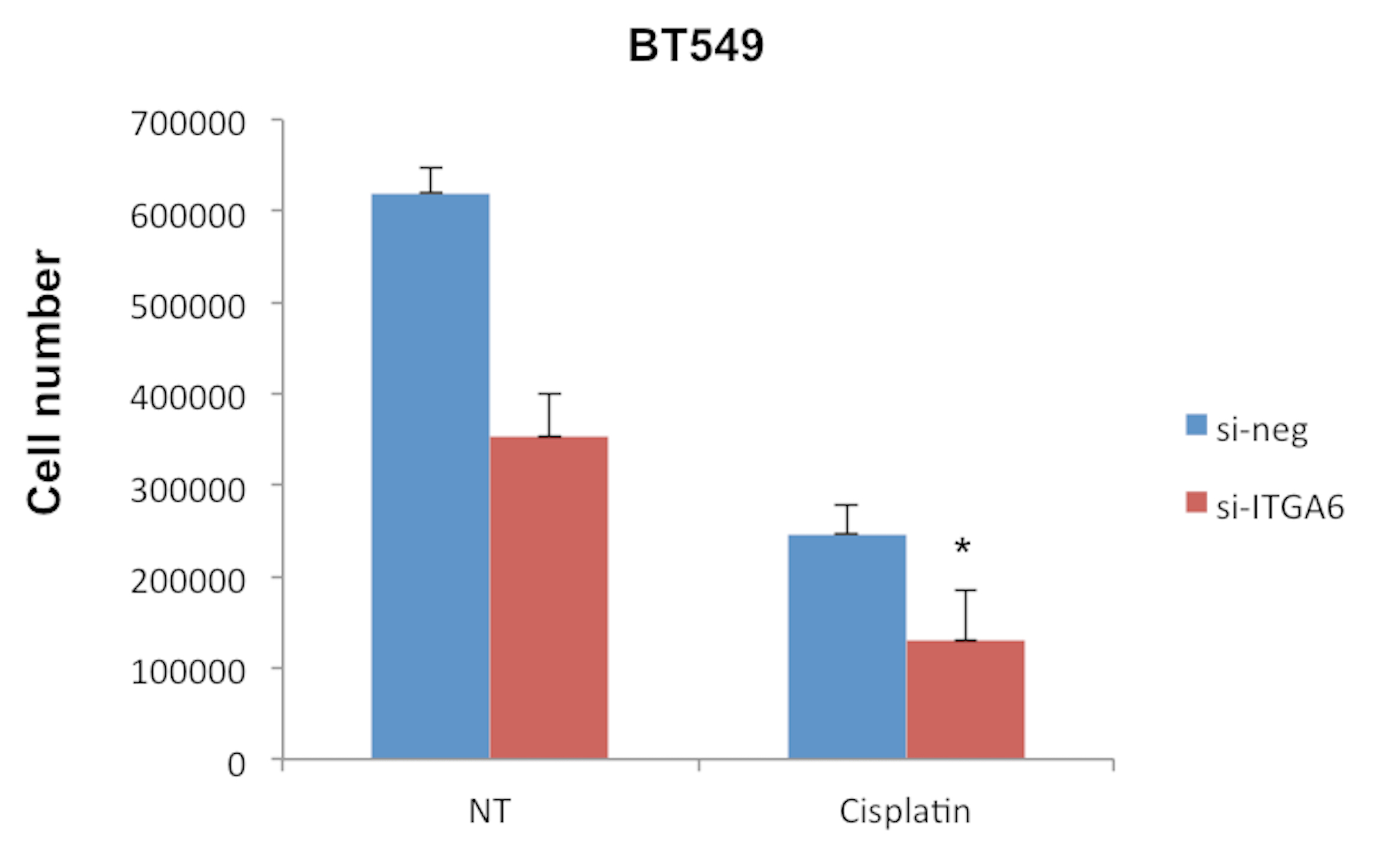

Supplement: Supplementary file 1 [file cancers-12-02261-s001.zip › Supplementary Figure 3.jpg]

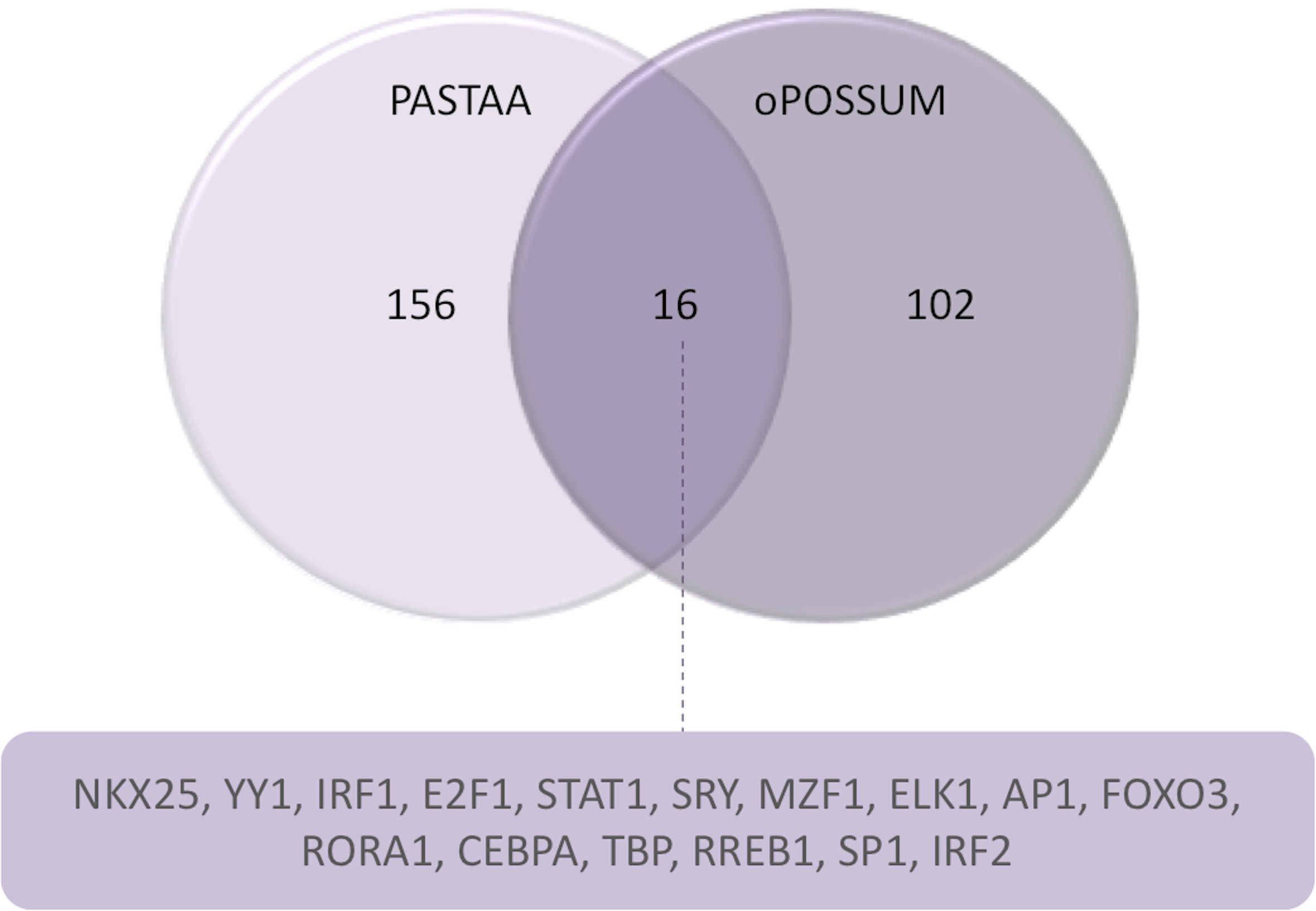

Supplement: Supplementary file 1 [file cancers-12-02261-s001.zip › Supplementary Figure 4.jpg]

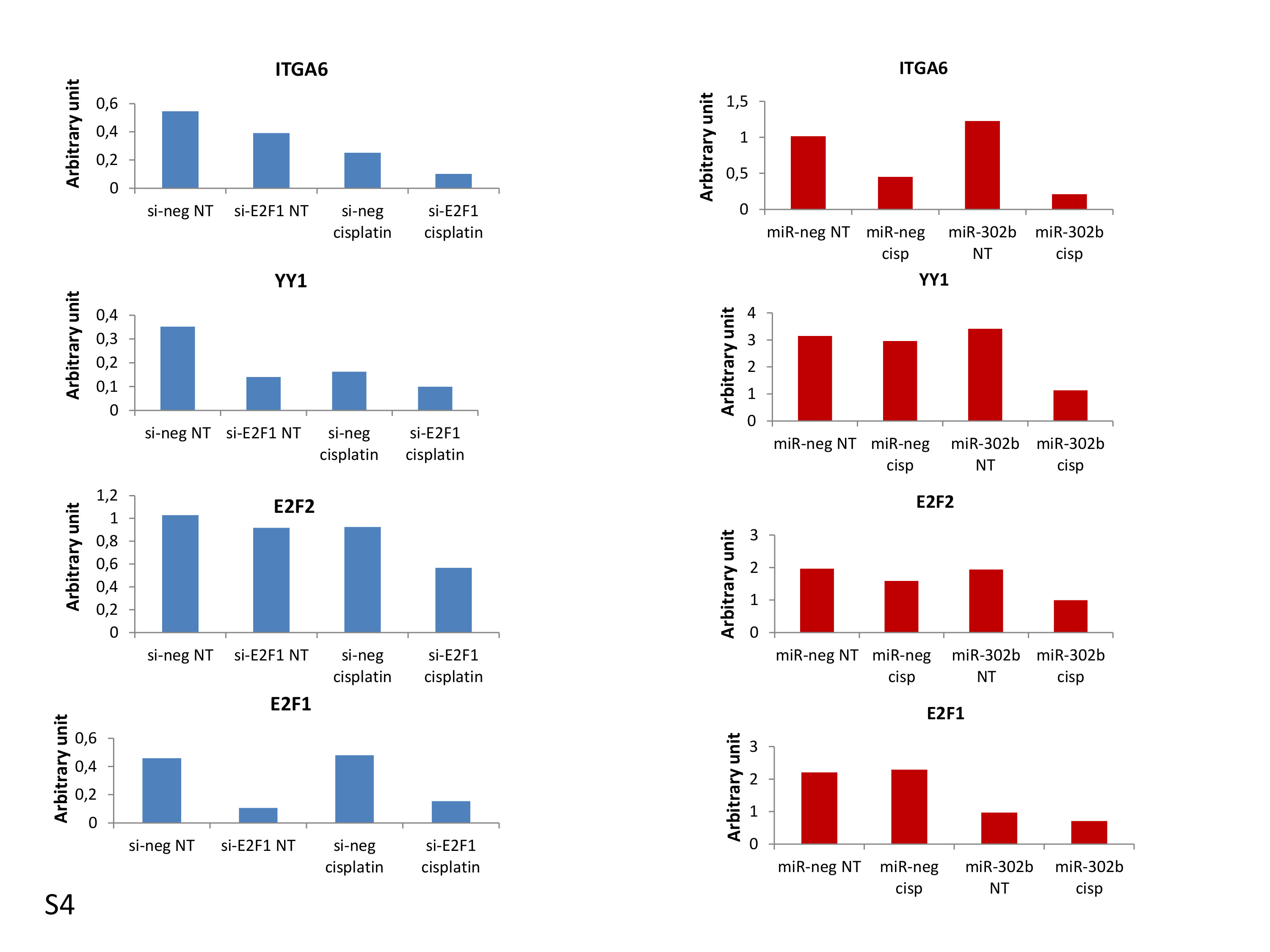

Supplement: Supplementary file 1 [file cancers-12-02261-s001.zip › Supplementary Figure 5.jpeg]

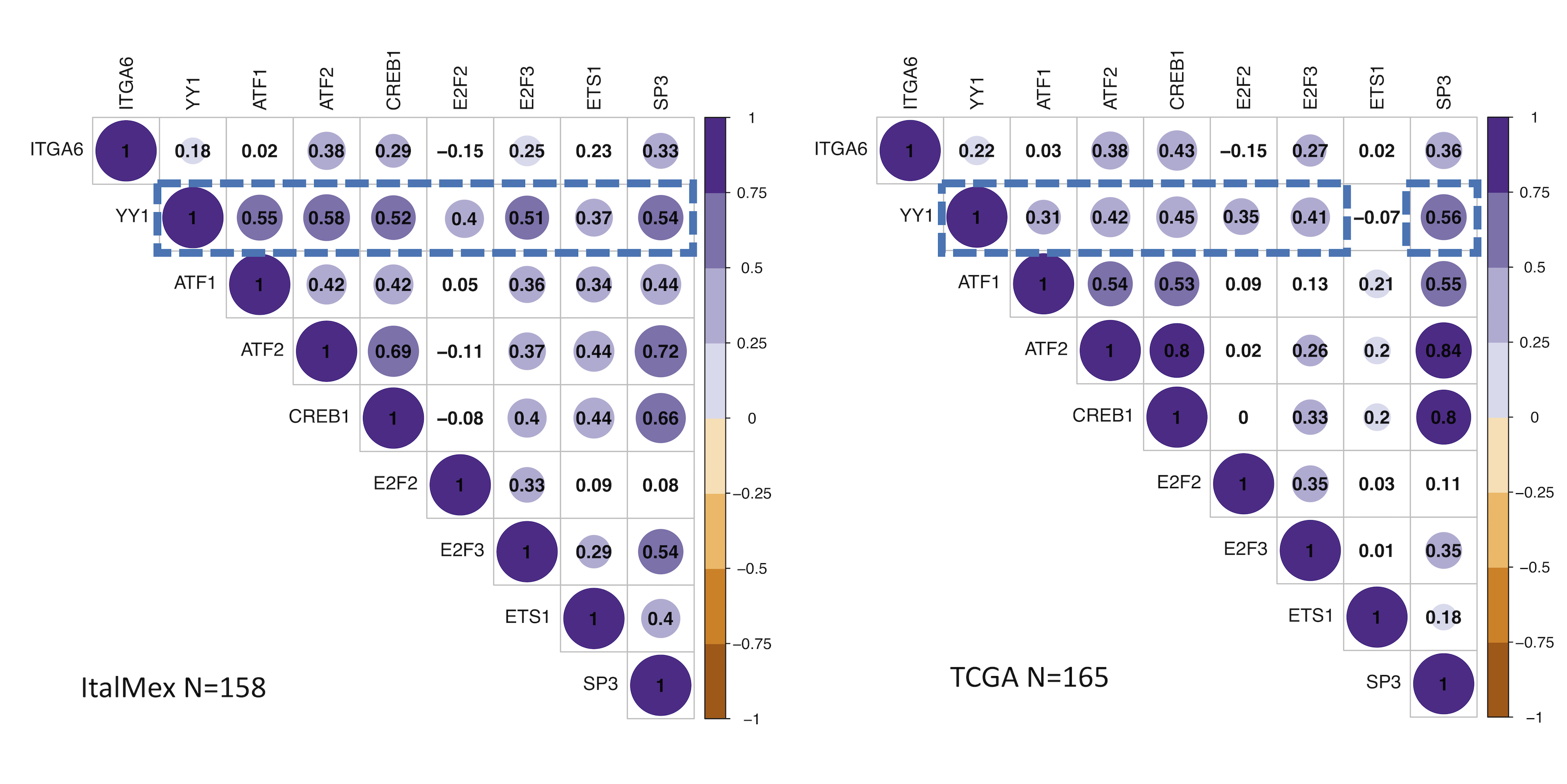

Supplement: Supplementary file 1 [file cancers-12-02261-s001.zip › Supplementary Figure 6.jpg]

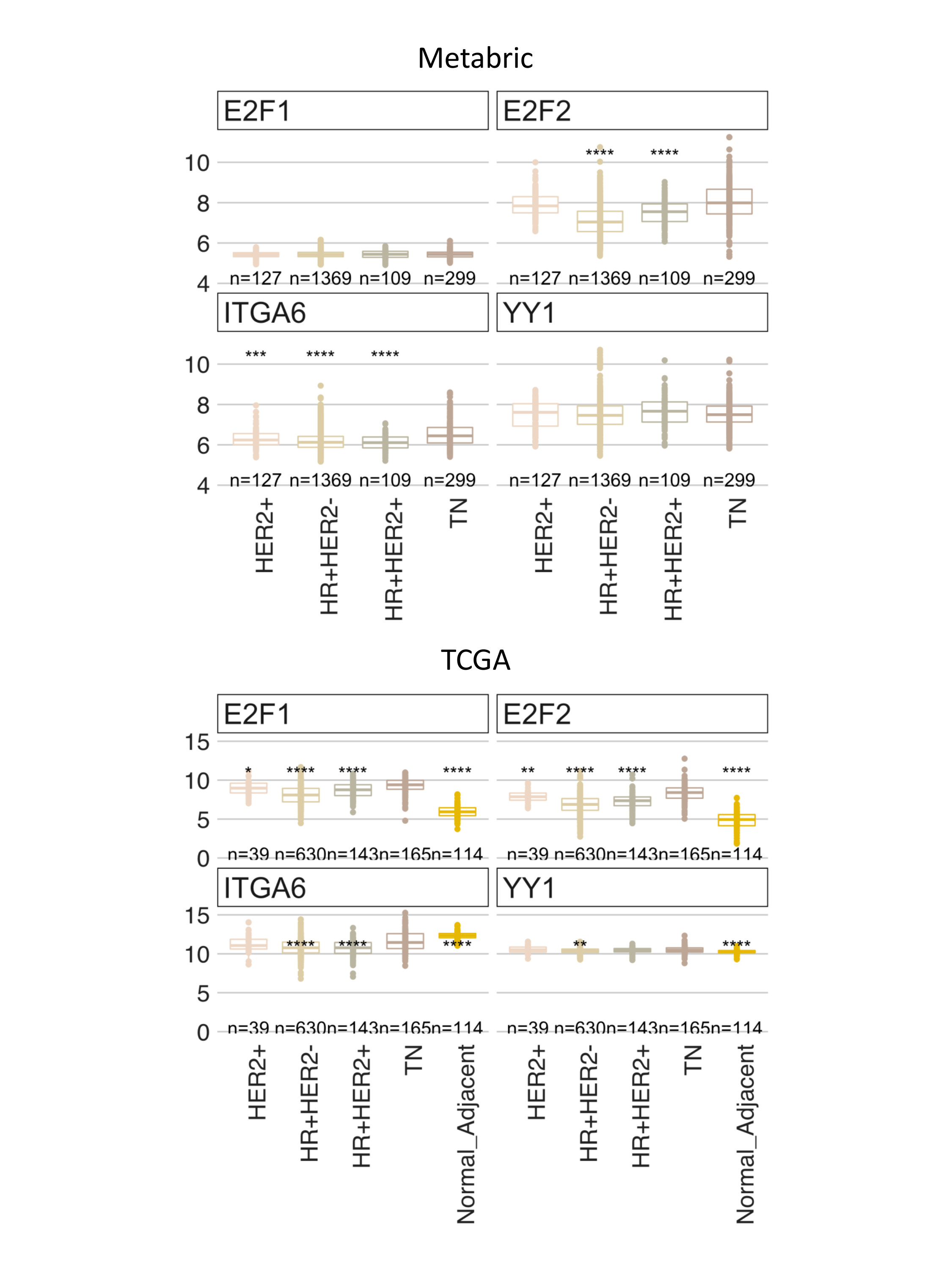

Supplement: Supplementary file 1 [file cancers-12-02261-s001.zip › Supplementary Figure 7.jpeg]
